# Structural Capsidomics of Single-Stranded DNA Viruses

**DOI:** 10.3390/v17030333

**Published:** 2025-02-27

**Authors:** Mario Mietzsch, Antonette Bennett, Robert McKenna

**Affiliations:** Department of Biochemistry and Molecular Biology, College of Medicine, Center for Structural Biology, McKnight Brain Institute, University of Florida, Gainesville, FL 32610, USA; rmckenna@ufl.edu

**Keywords:** ssDNA, capsid, *Parvoviridae*, *Microviridae*, *Circoviridae*, *Geminiviridae*, *Nanoviridae*, *Anelloviridae*, CRESS-DNA viruses

## Abstract

Single-stranded DNA (ssDNA) viruses are a diverse group of pathogens with broad host range, including bacteria, archaea, protists, fungi, plants, invertebrates, and vertebrates. Their small compact genomes have evolved to encode multiple proteins. This review focuses on the structure and functional diversity of the icosahedral capsids across the ssDNA viruses. To date, X-ray crystallography and cryo-electron microscopy structural studies have provided detailed capsid architectures for 8 of the 35 ssDNA virus families, illustrating variations in assembly mechanisms, symmetry, and structural adaptations of the capsid. However, common features include the conserved jelly-roll motif of the capsid protein and strategies for genome packaging, also showing evolutionary convergence. The ever-increasing availability of genomic sequences of ssDNA viruses and predictive protein modeling programs, such as using AlphaFold, allows for the extension of structural insights to the less-characterized families. Therefore, this review is a comparative analysis of the icosahedral ssDNA virus families and how the capsid proteins are arranged with different tessellations to form icosahedral spheres. It summarizes the current knowledge, emphasizing gaps in the structural characterization of the ssDNA capsidome, and it underscores the importance of continued exploration to understand the molecular underpinnings of capsid function and evolution. These insights have implications for virology, molecular biology, and therapeutic applications.

## 1. Introduction

Single-stranded DNA (ssDNA) viruses are a diverse set of viruses belonging to group II of the Baltimore classification [1]. They are among the smallest viruses but infect a wide variety of hosts, including bacteria, archaea, plants, protists, fungi, invertebrates, and vertebrates, and they are thus adapted to a broad range of environments and organisms [2,3]. Currently, the International Committee on Taxonomy of Viruses (ICTV) recognizes 35 virus families with ssDNA genomes (based on the 2023 release: https://ictv.global) (Table 1). Their small genomes typically range from ~1 to 10 kb and encode a limited number of essential proteins required for genome replication, packaging, and capsid formation [4]. The vast majority of the ssDNA virus families have circular genomes, and only a few have linear genomes, having hairpin-like secondary structures at both ends [5]. Additionally, a few families have multipartite genomes, where the viral genome is divided into multiple segments [6]. For genome replication, the circular ssDNA viruses utilize a rolling circle mechanism, while the linear viruses use a rolling hairpin replication mechanism [4]. Following genome replication, the newly formed virions enclose the viral genome to protect it from degradation and to facilitate the transmission of the genetic material from the producing cell to the next host cell. In this process, the virions must overcome multiple biological and physical barriers, including transversing different environmental conditions, culminating in the specific attachment to a receptor on a permissive cell. Due to the wide range of hosts existing in diverse ecological niches, the virions have evolved to adapt to these different environments. Consequently, the morphology of the ssDNA virions varies between the different families (Table 1). While most ssDNA viruses are non-enveloped, there are exceptions, including members of the *Pleolipoviridae,* whose virions are an assemblage of flexible membranous vesicles that lack a rigid capsid, nucleocapsid, and even nucleoproteins [7]. In contrast, viruses of the *Finnlakeviridae* have a capsid but possess an internal lipid membrane [8]. Furthermore, among the non-enveloped virions, members of the families *Inoviridae*, *Paulinaviridae*, *Plectroviridae*, and *Spiraviridae* display flexible filamentous particles with helical symmetry [9,10,11,12], while the remaining 28 virus families possess icosahedral capsids (Table 1).

These non-enveloped, icosahedral capsid architectures will be the focus of this review article. Their structural characterization has been ongoing for more than 30 years, initially using X-ray crystallography and, more recently, cryo-electron microscopy (cryo-EM). To date, structural information on the icosahedral capsids is available for eight of the ssDNA families. However, advances in DNA sequencing technologies in the last decade have resulted in a rapid increase in the number of newly identified virus families. As a result, virus families with structural data are currently in the minority. While many of these new viruses are under-studied and often only sequence information is available, new predictive structural tools such as AlphaFold [13] can be used to compare these newcomers to existing capsid structures.

**Table 1:** Overview of the ssDNA virus families and their characteristics.

Realm	Phylum	Virus Family	Virion Morphology	Genome	Hosts
*Monodnaviria*	*Hofneiviricota*	*Inoviridae*	Non-enveloped, filamentous	circular	Bacteria
		*Paulinaviridae*			
		*Plectroviridae*			
	*Phixviricota*	*Microviridae*	Non-enveloped T = 1 icosahedral capsid	circular	Bacteria
	*Commensaviricota* *	*Anelloviridae*			Invertebrates, Vertebrates
	*Cossaviricota*	*Bidnaviridae*	T = 1 icosahedral capsid + additonal capsid?	linear, segmented	Invertebrates
		*Parvoviridae*	Non-enveloped T = 1 icosahedral capsid	linear #	Invertebrates, Vertebrates
	*Cressdnaviricota*	*Adamaviridae*	Non-enveloped T = 1 icosahedral capsid	circular #	Invertebrates, Vertebrates
		*Amesuviridae*		circular	Plants
		*Anicreviridae*			Invertebrates, Vertebrates
		*Circoviridae*			Invertebrates, Vertebrates
		*Draupnirviridae*			Invertebrates, Vertebrates
		*Endolinaviridae*			Protists
		*Genomoviridae*		circular #	Fungi, Plants, Invertebrates, Vertebrates
		*Geplanaviridae*		circular	Plants, Invertebrates, Vertebrates
		*Kanorauviridae*			Plants, Invertebrates, Vertebrates
		*Kirkoviridae*			Vertebrates
		*Mahapunaviridae*			Plants, Invertebrates, Vertebrates
		*Metaxyviridae*		circular, segmented	Plants
		*Nanoviridae*			Plants
		*Naryaviridae*		circular	Protists
		*Nenyaviridae*			Protists
		*Ouroboviridae*			Invertebrates, Vertebrates
		*Pecoviridae*			Vertebrates
		*Redondoviridae*			Vertebrates
		*Smacoviridae*			Invertebrates, Vertebrates
		*Vilyaviridae*			Protists
		*Geminiviridae*	Non-enveloped pseudoT = 1 icosahedral capsid	circular #	Plants, Invertebrates
		*Bacilladnaviridae*	Non-enveloped T = 3 icosahedral capsid	Circular	Protists, Invertebrates
		*Gandreviridae*			Invertebrates, Vertebrates
	*Saleviricota*	*Pleolipoviridae*	Enveloped, pleomorphic particles	circular	Archaea
*Varidnaviria*	*Preplasmiviricota*	*Finnlakeviridae*	pseudoT = 21d capsid with internal membrane		Bacteria
not assigned		*Alphasatellitidae*	Satellite virus, genome packaged into capsids of the *Geminiviridae*		Plants, Invertebrates
		*Tolecusatellitidae*			Plants
		*Spiraviridae*	Linear, non-enveloped, and helical particles		Archaea

* *Anelloviridae* is suggested to be added to *Monodnaviria* and the phylum *Commensaviricota* [14]. # Some members of this family have segmented genomes.

## 2. Assembly of Icosahedral Capsids

**Figure 1:**
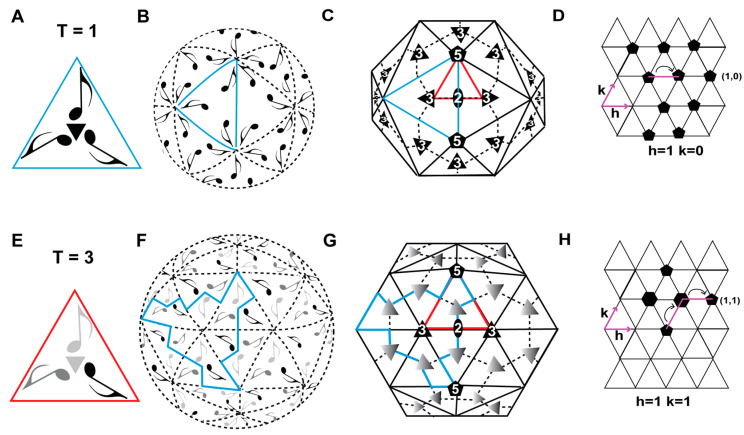
Symmetry-related interactions and triangulation number of icosahedral capsids. (**A**) Three identical musical notes (quaver) arranged with threefold symmetry (indicated by the black triangle), forming an equilateral triangle (blue open triangle). (**B**) Sixty quavers distributed with icosahedral symmetry representing the surface of the capsid. (**C**) T = 1 icosahedron with 20 triangular faces, where the center represents the 3-fold axis of symmetry, the edges of the triangle represent the 2-fold axis of symmetry, and the vertices represent the 5-fold axis of symmetry, with the viral asymmetric unit shown as a red open triangle. (**D**) Hexagonal plane of an icosahedral capsid, where the h and k vector planes (colored magenta) are 60° apart, with the location of each pentamer illustrated as a pentagon. (**E**) Quasi-equivalent arrangement of 3 quavers on the face of a triangle that may either be identical or non-identical, as represented by the different shades of gray. The red triangle represents the asymmetric unit of the T = 3 capsid and the small gray triangle the pseudo-3-fold axis. (**F**) Quasi-equivalent arrangement of 180 quavers on an icosahedral capsid surface. The triangular face of the T = 3 icosahedral capsid is colored blue. (**G**) T = 3 icosahedron with hexameric units representing the 3-fold axis, the edges of the triangle representing the 2-fold axis of symmetry, and the vertices representing the 5-fold access of symmetry. (**H**) Hexagonal plane of a T = 3 icosahedral capsid, where the h and k vector planes are 60° apart, with the location of each pentamer illustrated as a pentagon and a hexamer represented as a hexagon. The icosahedral 2-, 3-, and 5-fold axes are represented as an oval, triangle, and a pentagon, respectively.

The definition of an icosahedral capsid is founded on the historical description of platonic solids with point group symmetry operators of regular polygons, i.e. tetrahedron, cube, octahedron, dodecahedron, and icosahedron. An icosahedron can be described as a solid made from 20 equilateral triangular faces, arranged with 5-fold and 2-fold symmetry operators, with each equilateral triangle face possessing a 3-fold axis of symmetry (Figure 1A). An icosahedron is mathematically and genetically the most economical and efficient of all the platonic solids for the construction of a capsid. Multiple copies of an identical protein subunit encoded by a single gene can be arranged using the 5.3.2 symmetry operators to generate a completely enclosed solid with a large interior volume, which a virus can use to package its genome (Figure 1B). Within the icosahedral capsid, the placement of the capsid proteins (CPs) is defined by an “asymmetric unit”. The asymmetric unit (Figure 1, red triangle) describes the CP arrangements within the equilateral triangle (Figure 1, blue triangle), such that applying the 5- and 2-fold symmetry operators can generate the complete icosahedron. Therefore, the assembled icosahedron is composed of thirty 2-fold, twenty 3-fold, and twelve 5-fold axes (vertices) of symmetry. The smallest number of CPs that can assemble into an icosahedral capsid is sixty, and the simplest icosahedron is composed of twelve pentamers (each consisting of five CPs) (Figure 1C).

The first icosahedral virus capsid structures, determined by X-ray crystallography, were tomato bush stunt virus (TBSV) and southern bean mosaic virus (SBMV), both RNA viruses, provided a detailed description of the CP structural arrangement within the constraints of the icosahedral 5.3.2 symmetry operators [15,16]. This information formed the basis of the development of the theory of quasi-equivalence to describe the assembly of more complex icosahedral virus structures [17]. According to the quasi-equivalence theory, three or more CPs can be present in the viral asymmetric unit, which may be either identical or different proteins (Figure 1E), with each protein conformation leading to the formation of pentamers or hexamers. Furthermore, each CP can exhibit flexibility to conformationally switch to assemble the virus structure, with the local structural distortions maintaining the CP–CP interactions and contacts. The method of subdividing the icosahedral shell to describe these quasi-equivalent positions is called the triangulation numbering. The triangulation number (T) corresponds to the number of CPs in an icosahedron, which is 60 × T. The triangulation number also corresponds to the sub-triangulation of an icosahedral capsid into a planar hexagonal net, in which 12 uniformly spaced hexagons are substituted to pentamers (pentons), and their positions were defined by Caspar and Klug [17] as T = h^2^ + hk + k^2^, where h and k are positive integer numbers, representing the vector distance between each pentamer or 5-fold axis on each capsid (Figure 1D,H). Previously, three classes of icosahedrons were described [18]. The first class includes capsids with h ≥ 1 and k remaining at zero, such as the T = 1 capsids with h = 1 and k = 0. Other possible triangulation numbers for this class are 4 (h = 2), 9 (h = 3), 16 (h = 4), 25 (h = 5), etc. In the second class, the h and k values are identical, allowing for the triangulation numbers of 3 (h and k = 1), 12 (h and k = 2), 27 (h and k = 3) etc. The third class is also called the skew class, where the h and k values are ≥1 but not identical. Permissible triangulation numbers for this class include 7 (h = 2 and k = 1), 13 (h = 3 and k = 1), 19 (h = 3 and k = 2), and 21 (h = 4 and k = 1). However, the individual triangulation numbers for this class can also be achieved by switching the h and k values, in the case of a T = 21 capsid, either h = 4 and k = 1 or h = 1 and k = 4. These represent left-handed (*laevo* (l)) and right-handed (*dextro* (d)) enantiomorphic configurations of the icosahedrons [18].

More recently, some viruses have been identified that violate the above rules described by Caspar and Klug [17], such as capsids with 110 CPs, with two incomplete pseudo T = 1 capsids (each consisting of 55 CPs) to form a fused “two-headed” gemini capsid. Also, capsids assembled with 120 CPs have been observed that are referred to as pseudo-T = 2 icosahedrons. However, they could also be considered as T = 1 capsids with 60 dimeric CPs that originated by gene duplications [19]. Similarly, gene duplications within the CPs are also observed in capsids with higher triangulation numbers, resulting in the hexamers being composed of three dimeric CPs instead of six (e.g., pseudo-T = 21) [20]. To date, for the ssDNA viruses T = 1, T = 3, and pseudo-T = 21d icosahedral capsids have been described [20,21,22].

### 2.1. The Assembly of T = 1 Capsids

**Figure 2:**
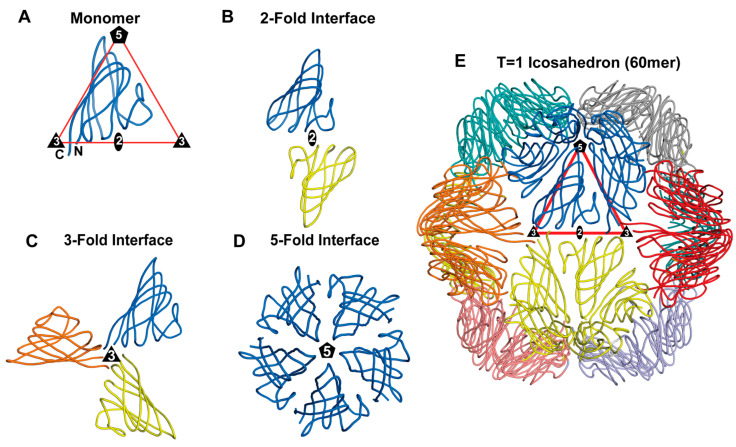
Symmetry-related interactions of the T = 1 icosahedral capsid of FBNSV. (**A**) Cartoon representation of a CP monomer. The N- and C-termini are labeled, and the approximate position of the icosahedral 2-, 3-, and 5-fold axes are shown. (**B**) The dimer, (**C**) trimer, and (**D**) pentamer interfaces are depicted. (**E**) The 60mer with the viral asymmetric unit is shown in the red triangle, as in Figure 1. The icosahedral 2-fold, 3-fold, and 5-fold axes are represented as an oval, a triangle, and a pentagon, respectively.

The assembly of the capsid is key in the virus lifecycle, and this process is governed by two principles: (i) specificity, where the encoded CPs have to fold into a defined structure to facilitate the appropriate CP–CP interactions at the 2-, 3-, and 5-fold symmetry interfaces to form the icosahedron via self-assembly or by utilization of a scaffold protein; and (ii) genetic economy, because the genomes are small and have limited coding capacity. Capsids are assembled by repeated usage of the same or few CPs rather than many different proteins. In the case of the T = 1 capsids, the simplest icosahedral arrangement, sixty CPs will interact via thirty 2-fold interfaces, twenty 3-fold interfaces, and twelve 5-fold interfaces (Figure 2).

**Figure 3:**
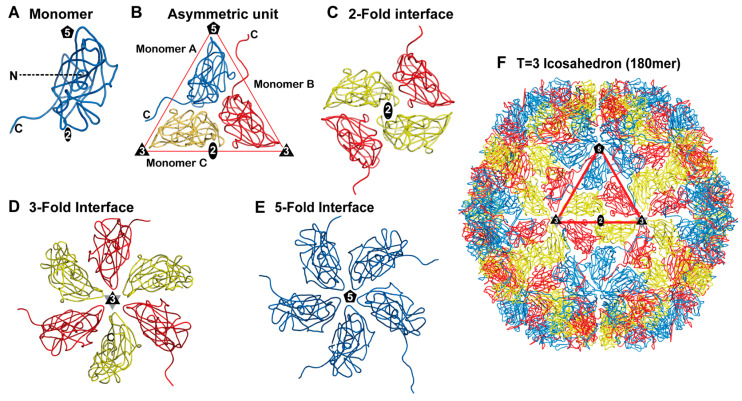
Symmetry-related interactions of the T = 3 icosahedral capsid of CtenDNAV-II. (**A**) Cartoon representation of a CP monomer. The N- and C-termini are labeled as N and C respectively, and the approximate position of the icosahedral 2-fold, 3-fold, and 5-fold axes are shown. (**B**) The asymmetric unit, (**C**) dimer, (**D**) trimer, and (**E**) pentamer interfaces are depicted. (**F**) The 180mer with the viral asymmetric unit is shown in the red triangle. The icosahedral 2-fold, 3-fold, and 5-fold axes are represented as an oval, a triangle (black and gray), and a pentagon, respectively.

Currently, the smallest known ssDNA virus T = 1 capsid structure is of Faba bean necrotic stunt virus (FBNSV), which will be used as an example for T = 1 icosahedral assembly (Figure 1E). The asymmetric unit contains one CP (Figure 2A) that interacts with other CPs at the symmetry-related 2-, 3-, and 5-fold interfaces. These interactions formed during the capsid assembly determine the physical properties of the capsid, namely the rigidity, flexibility, and stability, and they are likely associated with the number of interactions and the buried surface area of the interface [23]. The calculated buried surface area and number of interactions at the 2-fold (~400 Å^2^, 2 interactions) (Figure 2B) and 3-fold (~400 Å^2^, 2 interactions) interfaces are significantly less than what is observed for the 5-fold interface (~1000 Å^2^, 14 interactions) (Figure 2D) [24]. The calculated buried surface area for the formation of the symmetry-related interface is a measure of the association strength of the interface and likely determines the assembly pathway for these viruses. Mutagenesis of residue S88 and S87 (located at the 2-fold interface) of the FBNSV CP reveals a significant reduction in the number of assembled capsids concomitant with the increased appearance of pentameric capsomers [24]. Comparative analysis of the buried surface area for φX174 (T = 1) and maize streak virus (MSV), which has a pseudo-T = 1 capsid structure, also showed a significantly higher buried surface area and number of interactions for the 5-fold symmetry-related interface when compared to the 2-fold and 3-fold interfaces [23,25]. Pentameric intermediates have been isolated and characterized as crucial steps in the assembly of both these viruses [26,27].

### 2.2. Assembly of T > 1 Capsids

The capsid architecture of icosahedral viruses greater than 30 nm in diameter and with T > 1 symmetry can be described by the theory of quasi-equivalence (as described above). One such ssDNA virus with T = 3 icosahedral symmetry is *Chaetoceros tenuissimus* DNA virus type II (CtenDNAV-II) [21]. The CtenDNAV-II capsid is composed of 180 copies of quasi-equivalent CPs (Figure 3A,B).

In the CtenDNAV-II capsid, the viral asymmetric unit contains three CPs that are arranged with pseudo-3-fold symmetry (Figure 3B). Monomers A and B are almost identical, with amino acid 64–371 observed, while for monomer C residues 60–365 and 378–384 were observed, with some regions exhibiting alternative conformations compared to monomers A and B. The buried surface area and number of interactions of the CPs within the viral asymmetric unit are greater than all the other symmetry-related interactions (Figure 3B–E). Both the 2-fold and 3-fold interfaces utilize two structurally divergent CPs to generate the interface, specifically monomer C and B, while the 5-fold interface is generated by five copies of monomer A. Collectively, the 5-fold interactions and buried surface of CtenDNAV-II are less than that calculated for the other symmetry-related interactions (Figure 3C–E). It has been suggested that CtenDNAV-II may have emerged from ssRNA nodavirus-like capsids [21]. However, the assembly mechanism for CtenDNAV-II is not fully understood.

**Figure 4:**
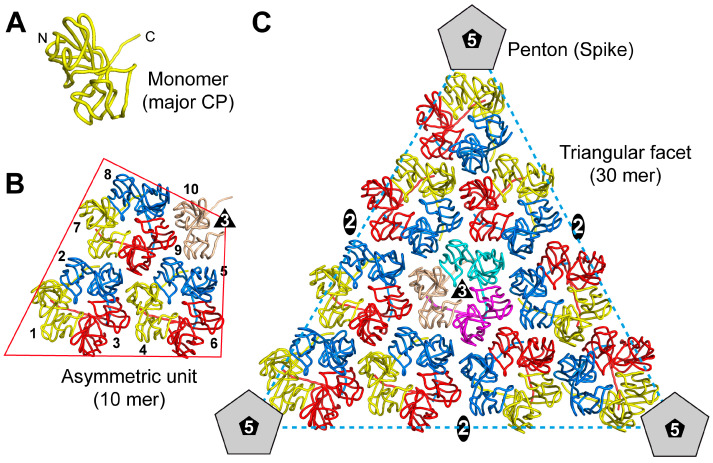
Symmetry-related interactions of a pseudo-T = 21 d icosahedral capsid. (**A**) Cartoon representation of the major CP monomer. The N- and C-termini are labeled. (**B**) The asymmetric unit composed of ten CPs is shown. Three CPs (yellow, blue, red) form a pseudo-hexameric unit. The tenth CP (salmon) of the asymmetric unit is unpaired but interacts with two additional asymmetric units (cyan and magenta) to form the central (**C**) pseudo-hexameric unit within the triangular facet of the pseudo-T = 21 d capsid. The icosahedral 2-fold, 3-fold, and 5-fold axes are indicated. At the 5-fold axis, the penton is formed by five copies of the minor CP.

The other ssDNA capsids that have had their capsid structure determined, exhibiting a triangulation number > 1, are pseudo-T = 21d capsids. Their viral asymmetric unit contains ten copies of the major CP, with nine of them forming three separate pseudo-hexamers or trimers (Figure 4). The remaining “unpaired” CP interacts with the “unpaired” CPs of two other asymmetric units to form the triangular facet of the pseudo-T = 21d capsid with a total of ten pseudo-hexamers. The assembled capsid is therefore composed of 10 × 3 × 20 = 600 major CPs. At each of the twelve 5-fold axes, a penton is formed by five copies of the minor CP (12 × 5 = 60 minor CP), bringing the total number of CPs for the capsid to 660 [20]. This morphology is also found in the dsDNA Pseudoalteromonas phage PM2. The capsid assembly has been suggested to be driven by the interaction of the triangular facets, described above, with each other and the internal membrane, until a nearly closed shell is formed. At the end, the 5-fold penton spikes are incorporated to complete the capsid [20].

## 3. Virus Families with Determined Capsid Structures

### 3.1. Parvoviridae

**Figure 5:**
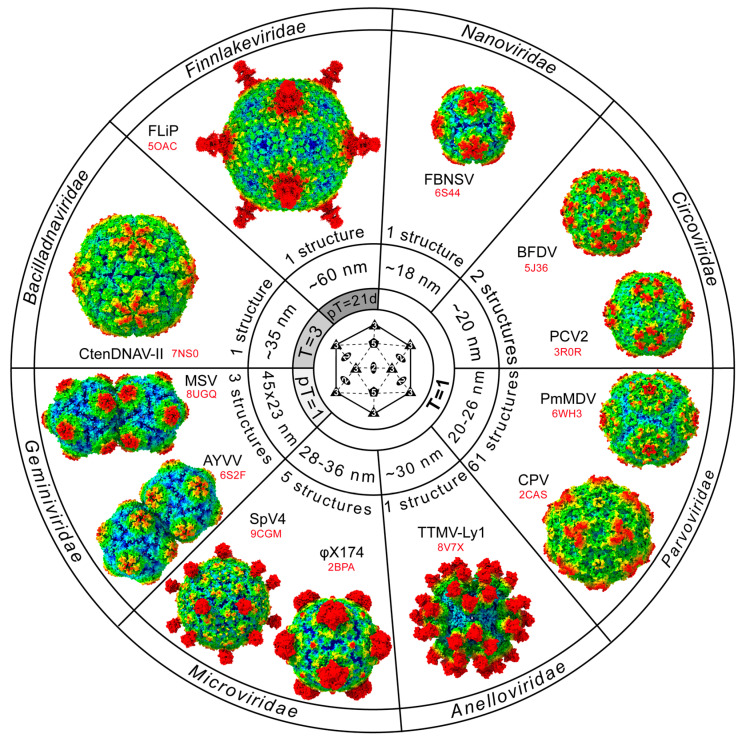
Overview of ssDNA virus families with determined capsid structures. Shown are 1–2 capsid structures per virus family. Each capsid is radially colored (blue to red) according to the distance to the center of the particle (other than the *Geminiviridae*, which are radially colored for each of the two incomplete half capsids). The number of determined capsid structures within each family, their approximate capsid size, their triangulation number, and the PDB-ID (in red) are provided. All capsid images are oriented as indicated by the symmetry diagram shown in the central circle of the wheel, centered down the icosahedral 2-fold axis. In a clockwise direction, starting at 1 o’clock, the viruses are as follows: FBNSV: faba bean necrotic stunt virus, BFDV: beak and feather disease virus, PCV: porcine circovirus, PmMDV: *Penaeus monodon* metallodensovirus, CPV: canine parvovirus, TTMV: torque teno mini virus, SpV: spiroplasma virus, AYVV: ageratum yellow vein virus, MSV: maize streak virus, CtenDNAV: *Chaetoceros tenuissimus* DNA virus, and FLiP: flavobacterium-infecting, lipid-containing phage. pT: pseudo-T.

The first capsid structure of a ssDNA virus was determined in 1991 for canine parvovirus (CPV) of the *Parvoviridae* by X-ray crystallography to 2.8 Å resolution [28]. The capsid was shown to form a T = 1 icosahedron of ~255 Å in diameter with a pore at each icosahedral 5-fold axis, protrusions around the 3-fold axis, and deep depressions at the 2-fold axes (Figure 5). The CPV capsid is composed of two overlapping proteins, viral protein (VP)1 and VP2, where VP1 shares its C-terminus with VP2 but possesses a unique N-terminus (VP1u) of 138 amino acids (aa) [22]. Both proteins are incorporated into the capsid in an approximate 5:55 ratio. However, in the CPV capsid structure only the shared VP2 region (548 aa) was observed, whereas the flexible VP1u region was not and believed to be located in the capsid interior. Furthermore, the glycine-rich N-terminus of VP2 was also structurally less ordered and was later found to be cleaved by proteases in capsids with packaged genomes [29,30]. The VP1u domains located inside the capsid are externalized under specific conditions in the endosomes during the post-entry steps after host cell uptake, enabling the capsid’s escape from these endosomes and transport to the nucleus via the VP1u phospholipase A_2_ (PLA2) domain and nuclear localization signals [31,32].

To date, with 61 determined capsid structures of unique, natural isolates, the *Parvoviridae* is structurally the most well characterized ssDNA virus family [22]. Furthermore, numerous additional *Parvoviridae* capsid structures at different pH or buffer conditions, in complex with antibodies or receptors, in the presence or absence of packaged genomes or VP1, as well as capsids with introduced amino acid changes, have been determined to reveal specific capsid functions, which are not covered in this review [33,34]. While initially X-ray crystallography was the method of choice for capsid structure determination, all *Parvoviridae* capsid structures after 2016 have been determined by cryo-EM. The adoption of the cryo-EM method to determine structures has accelerated the structural studies of parvoviruses, as the number of cryo-EM structures determined in the last 8 years has already exceeded all the previous structures determined by X-ray crystallography from the 25 years before that by a ratio of ~3:1.

The viruses of *Parvoviridae* have linear ssDNA genomes ranging from ~4 to 6 kb and are divided into three subfamilies: *Parvovirinae*, *Densovirinae*, and *Hamaparvovirinae* [35]. However, the number of deposited capsid structures are not equally distributed between these subfamilies. While there are fifty-four structures for *Parvovirinae*, there are only four structures for *Densovirinae* and three structures for *Hamaparvovirinae* (Table S1). Within the *Parvovirinae*, the capsid structures determined to date are from 7 of the 10 genera, with the majority being assigned to *Dependoparvovirus* (26 structures), followed by *Proto*- (14 structures), *Boca*- (9 structures), *Ave*- (2 structures), and one each for *Amdo*-, *Tetra*-, and *Erythroparvovirus*. The high number of structures for *Dependoparvovirus* is due to the adeno-associated viruses (AAVs) being a member of this genera, which have important medical implications, as they are used as vectors for gene therapy applications [36,37]. Despite low amino acid sequence identities, as low as 13%, between members of the different genera, the overall capsid morphology, described above for CPV, is generally maintained within *Parvovirinae*, with only variations in the surface topology of the capsid. Unlike most viruses of the subfamily with two VPs, members of *Dependoparvovirus* and *Bocaparvovirus* have been shown to express three overlapping VPs, with VP3 being the major capsid protein and VP2 having an N-terminal extension shorter than VP1 [38]. While the VP1 or VP2 N-termini are believed to be located inside the capsid, the VP1u regions of the erythroparvoviruses, such as Parvovirus B19, are located outside the capsid and contain receptor-binding domains [39,40].

Members of the *Densovirinae* have been described to express up to four overlapping VP proteins. The four determined capsid structures belong to the genera *Blattambi*-, *Itera*-, *Protoambi*-, and *Scindoambidensovirus* [41,42,43,44]. Their capsids lack large protrusions and thus appear slightly smaller than the capsids of *Parvovirinae*. Even smaller are the three capsids of *Hamaparvovirinae*, assigned to the genera *Brevihamadensovirus* and *Penstyldensovirus*, as well as the unassigned *Penaeus monodon* metallodensovirus (PmMDV) [45,46,47]. These capsids are formed by a single CP and primarily display protrusions around the 5-fold symmetry axis (Figure 5).

### 3.2. Microviridae

Following the determination of CPV, the capsid of the bacteriophage φX174, of the *Microviridae*, was the second ssDNA virus capsid structure determined in 1992 by X-ray crystallography to 3 Å resolution [48]. The φX174 virion is composed of four CPs, named F, G, J, and H. The F-protein assembles the T = 1 icosahedral capsid shell with a diameter of ~28 nm. At the 5-fold axes, the G-protein forms 12 prominent spikes, extending the outer diameter to ~34 nm (Figure 5). The small basic J-protein is located on the interior side of the capsid and is involved in DNA packaging [49]. Both the G-protein and J-protein follow the icosahedral symmetry of the F-protein (60 copies each). However, the ‘pilot’ H-protein is only incorporated at 10–12 copies per virion [50]. The exact location of the H-proteins within the virion is not fully understood [51]

The *Microviridae* is divided into two subfamilies, *Bullavirinae* and *Gokushovirinae* [52]. Viruses of this family have circular genomes ranging from ~4 to 6 kb. The *Bullavirinae* are further subdivided into three genera, *Alphatrevirus*, *Gequatrovirus*, and *Sinsheimervirus*. The bacteriophage φX174 belongs to the latter genus. Furthermore, the capsid structures of the bacteriophages α3 and G4 have been determined by X-ray crystallography [53,54]. Their capsids are homologous to φX174. For the *Gokushovirinae*, two capsid structures have currently been determined, both by cryo-EM [55,56]. Unlike for the bacteriophages α3 and G4, the capsids of the Escherichia phage φEC6098 and the spiroplasma virus 4 (SpV4) vary from φX174. Both capsids lack a homologous G-protein and instead display a spike at the 3-fold symmetry axis (Figure 5), which is part of the VP1 itself (homolog to F-protein). However, they conserve the VP8 (J-protein homolog) in the capsid interior. The φEC6098 and SpV4 phages belong to the genera *Enterogokushovirus* and *Spiromicrovirus*, respectively. Additionally, a low-resolution structure of the chlamydia phage 2 capsid of the genus *Chlamydiamicrovirus* has been determined, which shares structural similarity with φEC6098 and SpV4 [56].

### 3.3. Circoviridae

The *Circoviridae* is the third ssDNA family where structural characterization of these viruses was initiated using X-ray crystallography. The family is divided into two genera: *Circovirus* and *Cyclovirus* [57]. Their circular ssDNA genome, which gave the family their name, ranges from 1.7 to 2.1 kb. Viruses of the genus *Circovirus* infect exclusively vertebrates, whereas viruses of the genus *Cyclovirus* infect vertebrates and invertebrates [57]. Two capsid structures of members in this family have been determined, beak and feather disease virus (BFDV) and porcine circovirus 2 (PCV2), both belonging to the genus *Circovirus* [58,59]. The circoviruses express a single CP. Their icosahedral T = 1 capsids have a diameter of ~20 nm and display protrusions around the 5-fold axes (Figure 5). One difference between these capsids is the most depressed region, which is located at the 2-fold axis in BFDV and at the 3-fold axis in PCV2. For BFDV, interlocking capsid pentamers forming disks were observed, indicative of pentamers utilized for capsid assembly [59].

### 3.4. Nanoviridae

Members of the *Nanoviridae* are plant viruses. Their genome is composed of eight different ~1 kb circular ssDNA segments, which are each packaged in separate capsids. The family is divided into two genera, *Babuvirus* and *Nanovirus* [60]. Currently, the only capsid structure determined for *Nanoviridae* is for faba bean necrotic stunt virus (FBNSV) by cryo-EM to 3.2 Å resolution [24]. The T = 1 icosahedral capsid is ~18 nm in diameter, making FBNSV the currently smallest ssDNA virus (Figure 5). The most prominent feature of the capsid are protrusions surrounding the 5-fold symmetry axis, whereas the 2- and 3-fold regions are characterized by depressions, lining the pentameric capsomers. During virus purification and the subsequent structural characterization, some particles were described to have “missing” pentamers due to limited interactions along the 2- and 3-fold interfaces. Genomic DNA was observed to interact with the capsid under the 5-fold axis. Overall, the FBNSV capsid has been described as structurally related to the “single” capsids of geminiviruses [24].

### 3.5. Anelloviridae

The *Anelloviridae* is a family of extremely diverse viruses found in a wide host range of animals with circular ssDNA genomes [61]. It is divided into 34 genera and has been suggested to be added to *Monodnaviria* and the phylum *Commensaviricota* [14] (Table 1). Recently, the first capsid structure of this family for TTMV-Ly1 was determined by cryo-EM to 2.8 Å resolution [62], which belonged to the genus *Betatorquevirus*. The T = 1 icosahedral capsid shell has a size of ~20 nm in diameter. However, extensive spike domains are located surrounding the 5-fold symmetry axes, increasing the outer diameter to ~32 nm (Figure 5). Previously, a low-resolution cryo-EM reconstruction of chicken anemia virus of the genus *Gyrovirus* was determined that matches the overall capsid features of TTMV-Ly1, despite their capsid proteins sharing only 12% sequence identity [63].

### 3.6. Geminiviridae

The *Geminiviridae* is a family of highly pathogenic viruses that infects a wide range of agricultural crops in the tropics and subtropics. They are transmitted from host to host by insect vectors, specifically those belonging to the homopterans [64]. The *Geminiviridae* is divided into 15 genera based on their host range, insect vector, and genome organization [65]. Their circular genomes of 2.5 to 5.2 kb can be either monopartite or bipartite and are encapsidated in unique twinned pseudo-T = 1 icosahedral capsids with dimensions of ~22 × 38 nm (Figure 5). To date, three geminivirus capsid structures have been determined by cryo-EM: ageratum yellow vein virus (AYVV) to a resolution of 3.3 Å, maize streak virus MSV-N[A] to a resolution of 3.2 Å, and African cassava mosaic virus (ACMV) to a resolution of 4.2 Å [25,66,67]. The gemini capsids are composed of 110 CP monomers that form two pseudo-T = 1 icosahedral heads. However, at the equatorial region of the capsids where the two heads are joined, a pentamer is missing on the incomplete icosahedrons [25,66]. The viral asymmetric unit for the gemini capsid is an 11mer. All CP monomers are identical; however, they differ in the length of the N-terminus that is ordered in the structure, which is also based on its location in the capsid. The structural arrangement of the CPs at the waist of the capsid diverges from other CPs of the capsid, facilitating the interaction of the two heads of the capsid. The two incomplete icosahedrons are twisted approximately 20° along the longitudinal axis to each other. The capsid structures revealed fitted polynucleotides, likely of their packaged genome, at the intra-pentamer symmetry-related interface. In addition to the gemini capsid, a small percentage (~5%) of single T = 1 capsids with strict icosahedral symmetry are observed for MSV-N[A], and the capsid structure has been determined to 3.7 Å resolution [25].

### 3.7. Bacilladnaviridae

The *Bacilladnaviridae* contains seven genera, with its members infecting eukaryotic algae. Their circular genomes range from ~4 to 6 kb and express a single CP [68]. Currently, CtenDNAV-II is the only capsid structure, determined to 2.4 Å resolution by using cryo-EM, and it belongs to the genus *Protobacilladnavirus* [21]. Its capsid is a T = 3 icosahedron with a diameter of ~37 nm that packages a genome of ~6 kb, which is partially double-stranded (dsDNA) [69]. However, different conformations of the CPs were observed within the asymmetric unit, particularly for their C-terminal tails [21]. The most prominent features of the CtenDNAV-II capsid are the star-shaped protrusions around the 5-fold axes and depressed regions at the 3-fold axes (Figure 5). Structural comparisons of the capsid indicated that this virus may have emerged from RNA viruses, such as *Nodaviridae*, that also display T = 3 symmetry rather than from other ssDNA viruses [70]. Furthermore, in the CtenDNAV-II capsid structure, parts of the packaged genome inside the capsid were observed to form an outer genome layer, which was modeled as 630 bp of dsDNA and suggested to be the partial dsDNA region of the CtenDNAV-II genome [21].

### 3.8. Finnlakeviridae

The *Finnlakeviridae* is a recently established virus family with a single genus: *Finnlakevirus* [71]. The virion structure of the Flavobacterium-infecting, lipid-containing phage (FLiP) was determined by cryo-EM to 4 Å resolution [8]. This virus infects the Gram-negative bacterium *Flavobacterium* sp. and has a circular ssDNA genome of ~9.2 kb. The virion possesses a pseudo-T = 21d icosahedral capsid of ~60 nm with a ~5 nm thick internal membrane. The major CPs assemble the majority of the shell of the virion around the membrane, with depressed regions at the icosahedral 3-fold axes (Figure 5). At the icosahedral 5-fold vertices, ~12 nm tall pentameric spikes are formed by the minor CPs.

Recently, the structure of the ssDNA virus φCjT23 was determined by cryo-EM (PDB-ID: 7ZZZ) to 4.1 Å resolution [20]. This phage has a circular ~7.6 kb genome, also infects *Flavobacterium* sp., and shares a highly similar virion morphology with FLiP despite having no significant sequence similarity between the major CPs. However, the shape of the spikes at the 5-fold axes differs between the two viruses. Unlike for FLiP, the spikes form mushroom-like protrusions in φCjT23 [20]. To date, φCjT23 has not been assigned to a virus family but is located in the genus *Ficleduovirus* within the realm of *Duplodnaviria*, which contains dsDNA viruses (https://ictv.global/taxonomy, accessed on 10 February 2025). The reason for this is that the capsids of both FLiP and φCjT23 show similar morphology to several dsDNA virus capsids of the PRD1–adenovirus lineage and were thus considered to be one of the last common ancestors between ssDNA and dsDNA viruses [20].

## 4. The Structure of the Capsid Building Blocks

**Figure 6:**
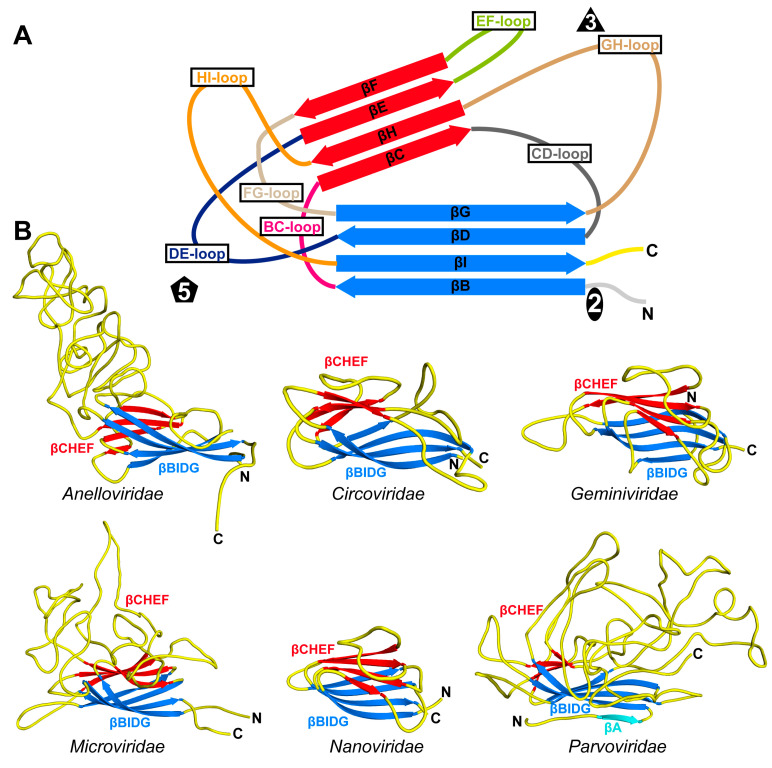
The jelly-roll motif in ssDNA viruses. (**A**) A depiction of a stylized capsid protein model with its jelly-roll motif, with the β-strands of the CHEF and BIDG sheets depicted in red and blue, respectively. The individual β-strands, the connecting surface loops, and the approximate positions of the icosahedral symmetry axes are labeled. (**B**) The capsid monomer structures of TTMV-Ly6 (*Anelloviridae*), PCV2 (*Circoviridae*), MSV (*Geminiviridae*), φX174 (*Microviridae*), FBNSV (*Nanoviridae*), and CPV (*Parvoviridae*).

When the first RNA virus capsid structures of TBSV and SBMV (both T = 3) [15,16], as well as satellite tobacco necrosis virus (STNV) [72] (T = 1), were compared in the early 1980s, Rossman et al. observed that their β-sheet regions exhibited structural homology with negligible amino acid sequence identity [73]. This fold, also known as the jelly-roll motif [74], is formed by two β-sheets, βBIDG and βCHEF, with the β-strands of each set of sheets running antiparallel to each other (Figure 6). The labeling of the β-strands from B to I is used for historical reasons based on the structures of TBSV and SBMV, which possess an additional βA strand prior to βB [15]. When the first ssDNA virus capsid structure was determined in 1991, a remarkedly similar arrangement of the central jelly-roll motif was observed in the CPV CP [28]. More than 30 years later, to date, all icosahedral ssDNA viruses, despite little to no sequence identity, utilize this fold for the core domain of their CPs. Additionally, the jelly-roll fold is also utilized in some viral non-capsid proteins and cellular proteins [75,76].

### 4.1. The Jelly-Roll Motif in ssDNA T = 1 Capsids

**Figure 7:**
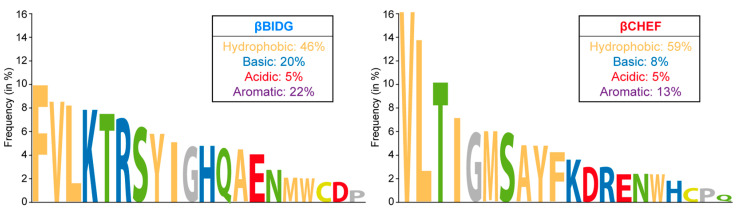
Amino acid occurrence frequency in the jelly-roll motif. The combined amino acid frequencies in βBIDG (**left**) and βCHEF (**right**) of TTMV-Ly6, PCV2, MSV, X174, FBNSV, and CPV are shown. Hydrophobic residues are colored orange, basic residues blue, acidic residues red, and polar residues green. Glycine and proline are colored gray.

The eight-stranded β-barrel in ssDNA viruses is typically arranged with the βB, βD, βF, and βH strands tangentially point towards the 5-fold axis of the capsid, whereas the βC, βE, βG, and βI strands point away (Figure 6). The inner βBIDG sheet forms the interior surface of the capsid and is often larger than the βCHEF sheet, especially in *Parvoviridae*. The βCHEF sheet is mostly buried within the capsids and partially contributes to the capsid interior and, in smaller capsids, both the interior and exterior surfaces (e.g., *Nanoviridae*). As mentioned above, no amino acid sequence identity in the jelly-roll motif between different ssDNA virus families has been observed. However, both sheets are generally very hydrophobic, with βCHEF having a higher percentage of hydrophobic residues (~60%) when compared to βBIDG (~45%) (Figure 7). In contrast, the βBIDG sheets have a higher frequency of basic and aromatic residues than βCHEF, which facilitates interactions with the viral DNA at the interior surface of the capsids.

**Figure 8:**
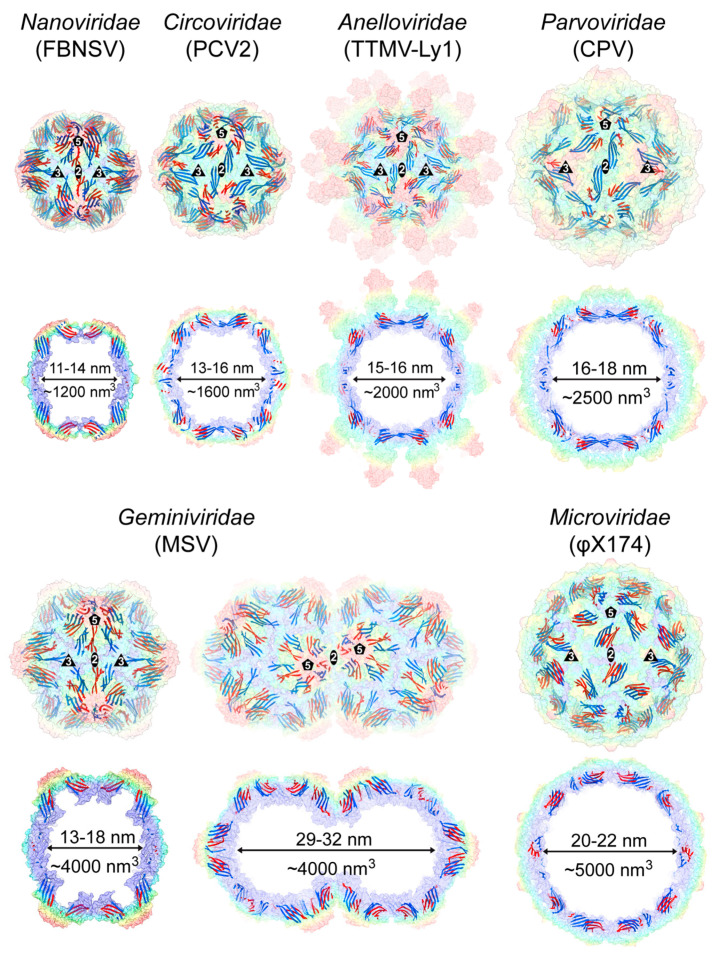
Positional location of the jelly-roll motif in the context of the capsid. Transparent radially colored (blue, green, yellow, to red, according to the distance to the center of the particle) surface representations for the capsids of the T = 1 ssDNA virus capsids. The βBIDG and βCHEF sheets are colored blue and red, respectively. The position of the 2-, 3-, and 5-fold symmetry axes are indicated. Below each capsid, a cross-section through the center of the capsid is shown, and the approximate diameter and volume inside the capsid are provided.

Despite the structural conservation of the core β-barrel fold of the ssDNA virus CPs, the jelly-roll motif is not positioned in the same way within their capsids. In the case of the capsids of the *Nanoviridae*, the βCHEF sheets are located at the 2-fold axis and extend towards the 5-fold axis, whereas the βBIDG sheets are located at the 3-fold axis and extend towards the 5-fold axis (Figure 8). The same arrangement is also observed for the *Geminiviridae* and, with some variation, for the *Microviridae*. In contrast, in the capsids of the *Circo*-, *Anello*-, and *Parvoviridae*, the βBIDG sheets are located at the 2-fold axis and extends towards the 5-fold axis. The βCHEF sheets are positioned between the βBIDG sheets and tend to be shorter for these viruses (Figure 8).

While the jelly-roll sheets are slightly curved, they were previously described as being arranged approximately tangential relative to the sphere of the capsid when only capsid structures of the *Parvo*- and *Microviridae* were available [77]. However, in the capsids of the *Nano*- and *Geminiviridae*, the βBIDG and βCHEF sheets are positioned more diagonally to the radius of the capsid (Figure 8). This positioning causes the protruding of the 5-fold region on these capsid surfaces, creating sub-pockets in the interior of the capsid. Within these interior pockets, additional densities have been observed in cryo-EM maps for both families, which were suggested to be the CP N-termini or the packaged genome [24,25].

### 4.2. The Surface Loops of ssDNA T = 1 Capsids

The total number of amino acids contributing to the jelly-roll motif for the ssDNA viruses of the different families is approximately the same (78 ± 8 aa). Between the β-strands, loops of variable lengths are inserted that contribute to the surface features of the capsids. These loops are often named based on the flanking β-strands, for example: the loop connecting the βB and βC strands is referred to as the BC-loop (Figure 6). Unlike the jelly-roll motif, the total number of residues within these loops can vary widely between the families. In the case of FBNSV, only ~38% of the CP accounts for the surface loops, whereas for φX174, 73% of the residues in the F-protein are within the surface loops (Table 2). Furthermore, among the surface loops for most viruses, there are usually one to two “dominating” loops that are significantly larger than the other loops. For the T = 1 ssDNA virus families with known structures, these are primarily the EF-, GH-, and HI-loops (Table 2).

The orientations of the β-strands generally place the BC-, DE-, FG-, and HI-loops near the 5-fold symmetry axes (Figure 6). In contrast, the remaining CD-, EF-, and GH-loops are positioned either near the 2-fold or 3-fold symmetry axes (Table 2). Additionally, the residues following the βI-strand often contribute to the capsid surface, while the residues preceding βB primarily reside in the interior of the capsid. The N-terminal amino acids of the CPs are often not observed to be structurally ordered, regardless of the structure determination method, i.e., either X-ray crystallography or cryo-EM.

Structural studies have highlighted that the surface loops of the capsids play important roles in the viruses’ life cycle. They mediate the interaction to specific cellular receptors on their target cells [78]. Several glycan and proteinaceous receptors have been identified for different viruses [79,80,81,82,83,84]. For some of these, the binding sites have been identified by determination of the capsid structure in complex with the receptor molecule [85,86,87,88]. Additionally, vertebrate immune systems produce antibodies against the viral capsid surface loops. Currently, for two ssDNA virus families, complex structures of the capsids with antibodies have been described [33,89].

### 4.3. The Architecture of T > 1 Capsids

**Figure 9:**
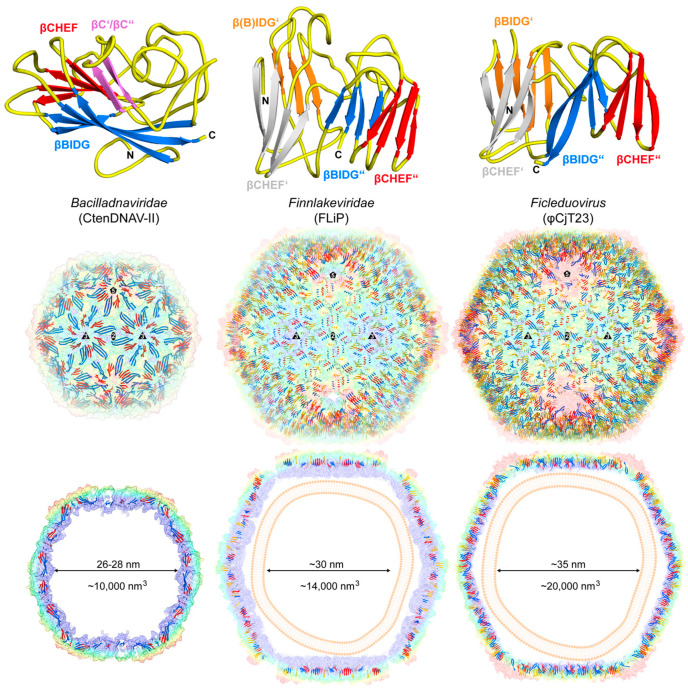
The jelly-roll motif in ssDNA T > 1 capsids. The capsid monomer structures of CtenDNAV-II (*Bacilladnaviridae*), FLiP (*Finnlakeviridae*), and φCjT23 (*Ficleduovirus*) are displayed. Their βBIDG (blue/orange), βCHEF (red/gray), and sheet extensions (magenta) are labeled. Transparent radially colored (blue, green, yellow, to red, according to the distance to the center of the particle) surface representations for the capsids are shown. The position of the 2-, 3-, and 5-fold symmetry axes are indicated. Below each capsid, a cross-section through the center of the capsid is shown, and the approximate diameter and enclosed volume of the capsid interior are provided.

The CPs of the ssDNA virus families with T = 3 or pseudo-T = 21d capsids also possess the jelly-roll motifs. In the T = 3 capsid structure of the *Bacilladnaviridae*, the βBIDG sheet is located on the interior surface of the capsid (Figure 9), similar to the T = 1 capsids (Figure 6). However, it is partially occluded by the 24 aa CP N-terminus, which is located under the βBIDG sheet. The βCHEF sheet is nearly completely buried within the capsid but is extended by two additional anti-parallel strands in the CD-loop. Due to their similar tangential positions within the capsid, the sheets follow the trend of the T = 1 capsids, with βCHEF being slightly more hydrophobic than the βBIDG sheet (56 vs. 50%) and with the latter containing more basic residues (8 vs. 14%). In the T = 3 capsids, the β-sheets are all approximately oriented from the pseudo-3-fold axis within the asymmetric unit (Figure 3B) towards the icosahedral 3- or 5-fold axes (Figure 9). Overall, the jelly-roll motif contributes to approximately half of the CP structure (Table 3). The dominating surface loops in this capsid are the EF- and GH-loops that are situated around the pseudo-3-fold region. In contrast, the shorter BC-, DE-, FG-, and HI-loops are located either near the icosahedral 3- or 5-fold axes of the capsid.

Unlike the T = 1 and T = 3 capsids, the pseudo-T = 21d capsid structures contain two jelly-roll motifs (Figure 9). In the capsid structure of FLiP in the family *Finnlakeviridae*, the β-strand B of the first jelly-roll motif was not modeled [8], potentially due to the overall lower resolution of the density map, but it is most likely located at the N-terminus. The second jelly-roll motif is positioned nearly parallel to the first in both FLiP and φCjT23, connected by a short linker sequence (Figure 9). Within the capsid, the jelly-roll motifs are arranged radially, resulting in the BC-, DE-, EF-, and HI-loops forming the outer surface of the capsids and the CD-, EF-, and GH-loops the inner surface of the capsids. The loops on the exterior surface are on average longer than those on the interior surface, with the DE- and FG-loops being the longest (Table 3). The jelly-roll motifs of the T = 21d capsids contribute ~38–46% to the total capsid protein and are hydrophobic, however, possibly because of their different arrangements and alternate evolutionary origin, they do not follow the trends observed for the T = 1 and T = 3 capsids, with ~54 and ~44% hydrophobic residues in the βBIDG and βCHEF sheets, respectively. The percentage of basic residues ranges from 11 to 16% for βBIDG and βCHEF.

### 4.4. Genome Packaging of the ssDNA Virus Capsids

One of the primary functions of the viral capsid is to package and protect the viral genome until the infection of a host cell and release it for replication. Generally, there are two strategies viruses use to encapsidate their genomes: (I) the viral genome is packaged within the capsid as it is being assembled; or (II) the viral genome packaging occurs after capsid assembly is completed [90]. For the latter, the initially empty capsid is often referred to as a procapsid, which may undergo conformational changes during the genome packaging process. For the ssDNA viruses with known capsid structures, strategy I has been suggested for the *Geminiviridae*, *Bacilladnaviridae*, *Finnlakeviridae*, and *Ficleduovirus* [20,21,91,92], whereas the *Microviridae* and *Parvoviridae* utilize strategy II [30,51,93,94]. Additionally, many ssDNA viruses possess highly basic peptide arms at their CP N-termini that are required for genome encapsidation [95,96,97].

**Figure 10:**
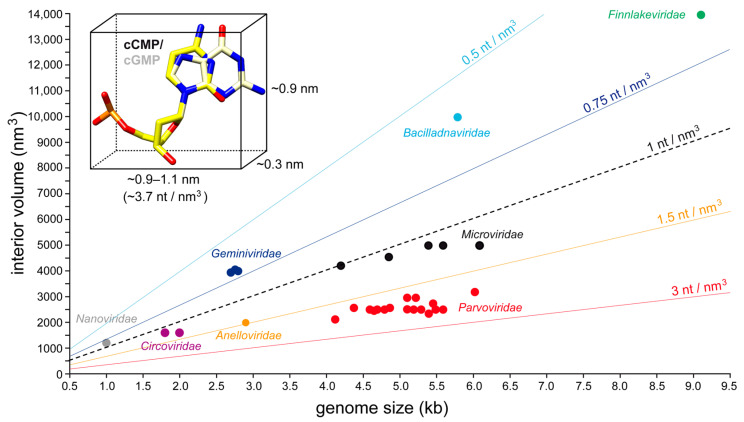
Genome packaging density of ssDNA virus capsids. Plotted are the available interior volumes of determined ssDNA virus capsids vs. the sizes of their genomes. The colored lines indicate various packing densities from 0.5 to 3 nt/nm^3^. For reference, the dimensions of a dCMP and dGMP nucleotide are provided.

The size of the capsid, specifically the available volume of the interior, limits the amount of ssDNA that can be packaged. Among the eight ssDNA virus families with determined capsid structures, the capsids of the *Nanoviridae* have the least available volume for packaging followed by the *Circoviridae* (Figure 8). Consequently, these two families have the smallest genome sizes, with ~1 or ~2 kb of ssDNA. Both families package their genome into the capsid at a density of approximately 1 nucleotide (nt)/nm^3^ (Figure 10). However, for the remaining families, the capsid and genome sizes do not correlate. While the capsids of the *Microviridae* also package genomes at ~1 nt/nm^3^, the capsids of the *Gemini*-, *Bacilladna*-, and *Finnlakeviridae* utilize the available space less efficiently, with a packing density of 0.5–0.75 nt/nm^3^. The packing density of the phage φCjT23 (Figure 10), with its 7642 nt genome [20], is even lower than 0.5 nt/nm^3^. Interestingly, these viruses with packaging densities of <0.75 nt/nm^3^ all belong to the viruses that form their capsids around the viral genome or lipid-enclosed genomes. In contrast, the *Anello*- and *Parvoviridae* package their genomes tightly into preassembled empty capsids at a density of ~1.5–3 nt/nm^3^ (Figure 10). At these values, the DNA is packaged close to the previously reported density of DNA of 1.7 g/cm^3^ or 3.13 nt/nm^3^ [98].

## 5. The ssDNA Capsidome

**Figure 11:**
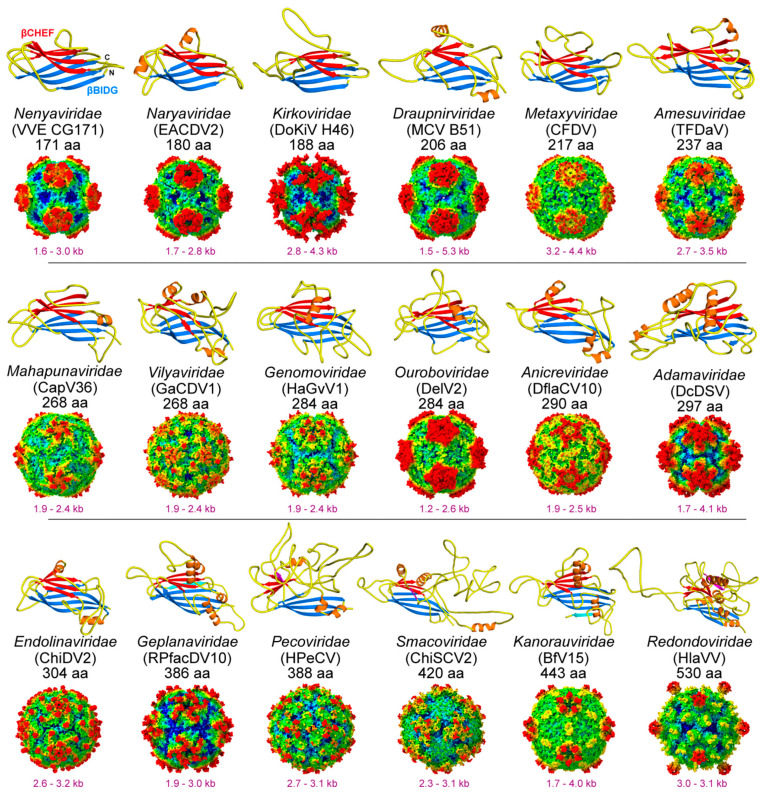
The capsids of the *Cressdnaviricota*. Shown are AlphaFold3 models of the viral CPs with the βBIDG sheet colored blue and βCHEF red. Alpha helices are colored orange. The ssDNA virus family, the selected virus, and the size of its CP are listed. Below each CP model, the corresponding generated assembled capsids with the genome size range are provided for each family. The capsids are radially colored (blue, green, yellow, to red, according to the distance to the center of the particle).

For the current 35 families of ssDNA viruses, the vast majority have either confirmed or are predicted to utilize some form of icosahedral capsid (Table 1). However, only eight of these families have experimentally determined structures and therefore, have confirmed their icosahedral geometry (Figure 5). This leaves many families without structural characterizations of their capsids. Most of these belong to the phylum *Cressdnaviricota*. The capsid proteins of this taxonomic group range from 171 to 530 aa (Figure 11). The smallest CPs belong to *Nenya*- and *Naryaviridae*, comparable to the capsid of FBNSV of the *Nanoviridae* (172 aa). At the other end of the spectrum, *Gandr*- and *Redondoviridae* have the largest CPs, with 468 and 530 aa, respectively.

In the absence of experimentally obtained structural information for these families, predictive protein folding tools such as AlphaFold [13] can be used to obtain insights into their capsid structures. In this review, for the remaining families without high-resolution data, AlphaFold3 was used, which successfully predicted the core jelly-roll motif in their CPs (Figure 11). Similar to the *Nanoviridae*, in the shorter CPs, the jelly-roll motif dominates the overall structure with only short loop insertions (Table 4). As a result, these proteins showed the highest similarity to FBNSV when superposed and, thus, likely possess T = 1 icosahedral capsids. While AlphaFold can predict the CP monomer structure, the exact placement of the jelly-roll motif within the 60mer capsid and the distance of the monomers to the center of the capsid are more problematic to predict and, thus, the arrangement of the virus with the highest structural overlap was used as a template for the placement of the CPs and the generation of an icosahedral capsid. Since the *Nano*-, *Circo*-, and *Geminiviridae* are members of the phylum *Cressdnaviricota*, one of these viruses was often used as a template, resulting in many of the new virus families of this phylum having capsids with dominant protrusions in the 5-fold region (Figure 11), similar to the previously determined capsid structures (Figure 5). These predictions will need to be verified experimentally. Nonetheless, the presence of surface loops with variable lengths generates unique capsids for these viruses.

**Figure 12:**
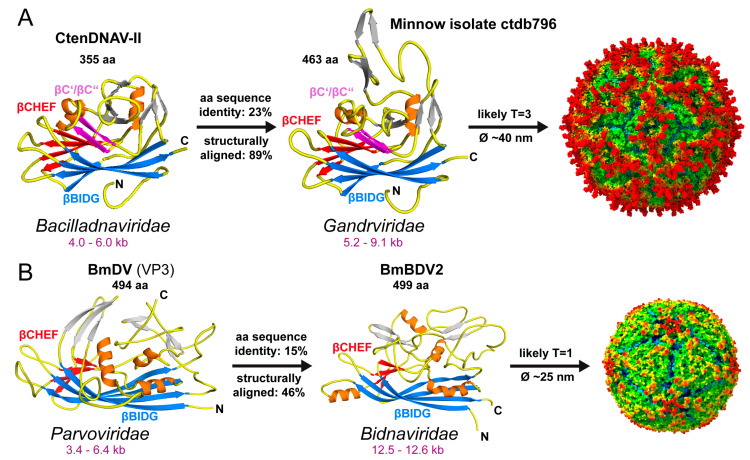
The capsids of the ssDNA virus families *Gandrviridae* and *Bidnaviridae*. (**A**) The CP monomers of CtenDNAV-II and minnow isolate ctdb796 of the *Bacilladnaviridae* and *Gandrviridae* are shown, respectively. The βBIDG sheet is colored blue, βCHEF red, βC’/ βC’’ magenta, other β-strands in gray, and the α-helices in orange. The percentages of amino acid sequence identity and structurally aligned residues are provided. A T = 3 capsid based on the *Gandrviridae* CP model is shown. (**B**) Depiction, as in A, of bombyx mori densovirus (BmDV) and bombyx mori bidensovirus 2 (BmBDV2), with a T = 1 capsid generated for BmBDV2. The capsids are radially colored, red for the capsid exterior, yellow followed by green for the body and blue extending to the capsid interior.

The capsids of the selected viruses of the *Peco*- and *Smacoviridae* may diverge from most viruses of the other *Cressdnaviricota*, with prominent 3-fold protrusions rather than 5-fold protrusions due to their long GH-loops (Table 4). Given the genome size range of ~1.5–5 kb, it is possible that these viruses possess T = 1 icosahedral capsids. In the case of the *Genomoviridae*, capsids of fusarium graminearum gemytripvirus 1 and diaporthe sojae circular DNA virus 1 were shown to assemble ~25 nm, likely T = 1 icosahedral, capsids by negative stain EM [99,100]. However, in absence of EM data for most families pseudo-T = 1 icosahedral capsids like the *Geminiviridae* or T = 3 capsids cannot be ruled out. One exception is the capsids of the Gandrviridae. The genomes of this family range from 5.2–9.1 kb. The AlphaFold model of the isolate ctb796, assigned to this family, shows high structural similarity (including the extended βCHEF sheet) to the CP structure of Cten-DNAV-II of the *Bacilladnaviridae* (Figure 12A). Thus, the capsids for this family are highly likely to be assembled into T = 3 icosahedral capsids with a diameter of ~40 nm with potential protrusions along the 2-fold axis towards the 5-fold axis.

Another ssDNA virus family currently without an experimentally determined capsid structure is the *Bidnaviridae*. This family is in the phylum *Cossaviricota* alongside the *Parvoviridae* (Table 1). The bidnaviruses are suggested to have originated from a parvovirus ancestor [101]. However, unlike the parvoviruses, the bidnaviruses have two linear ssDNA genome segments (each 6.0–6.5 kb), which are packed into separate capsids. Their capsids are predicted to have a similar jelly-roll CP but may differ significantly in their surface loops arrangements, as shown for the bombyx mori densovirus (BmDV) structure [42] and the bombyx mori bidensovirus (BmBDV2) AlphaFold model (Figure 12B). The resulting capsid for BmBDV2 shows a smooth-like surface with only small protrusions surrounding the 5-fold axes.

A possible way to group the capsids of the ssDNA virus families is based on their “dominant” loop insertions (Table 4). Group 1 consists of viruses with smaller CPs of ~300 aa or less and have their longest insertion in the EF-loop (Table 2). This group of viruses are comparable to the *Nanoviridae* and *Geminiviridae* capsids and would also include the *Nenya*-, *Narya*-, *Kirko*-, *Metaxy*-, *Amesu*-, *Mahapuna*-, *Genomo*-, *Ourobo*-, and *Adamaviridae*. The second group of viruses could be defined as those with smaller CPs and with the GH-loop being the dominant insertion loop, similar to the capsids of the *Circoviridae*, and it would further include the *Draupnir*-, *Vilya*-, *Anicre*-, and *Endolinaviridae*. For the virus families with larger CPs, such as the *Smaco*- and *Bidnaviridae*, the EF- and GH-loops dominate the CP structures (group 3), similar to the *Parvoviridae* (Table 2 and Table 4). The fourth group would also comprise virus families with larger CPs but with long CD-, EF-, and GH-loops, comparable to the *Bacilladnaviridae*, and it would also include the *Peco*- and *Gandreviridae*. This opens the question of whether the *Pecoviridae* have T = 3 capsids, despite their relatively small genome size (Figure 11). The remaining families cannot be placed in any of the four groups above. While the *Geplana*- and *Kanorauviridae* possess the combination of long CD- and EF-loops, the *Redondoviridae* have long loops in their BC-, DE-, EF-, GH-, and HI-loops (Table 4). Similarly, the capsids of the *Anelloviridae* are unique with their long HI-loops, as are those of the *Microviridae*, with their combination of long EF-, and HI-loops. The latter may not be surprising, as the *Microviridae* is the only ssDNA virus family with T = 1 or T = 3 capsids infecting bacteria identified to date. Interestingly, the viruses infecting plants all belong exclusively to the group of the *Nanoviridae-*/*Geminiviridae*-like capsids or the *Geplana*- and *Kanorauviridae* group.

## 6. The CP N-Termini

**Figure 13:**
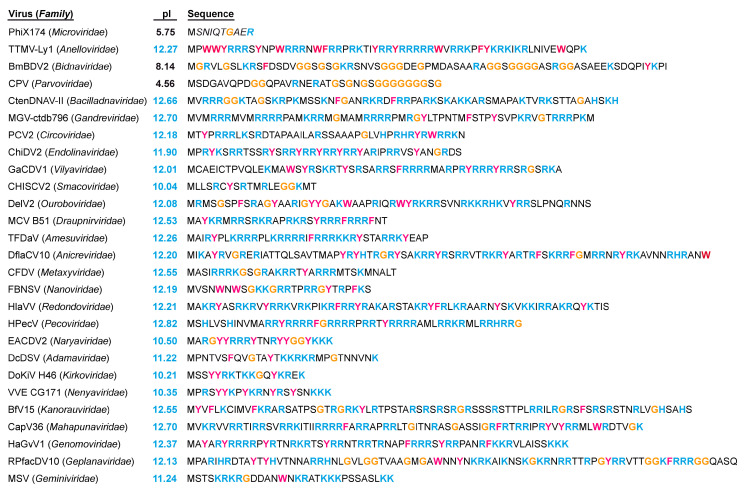
The N-termini of the ssDNA CPs with T = 1 and T = 3 capsids. The pI and amino acid sequence of the selected viruses are shown. Residues in blue indicate basic amino acids, those in magenta indicate aromatic residues, and those in orange indicate glycine.

Another characteristic of the ssDNA viruses is their CPs N-termini, which are not observed in many structures that have been previously determined, because they are either not structurally ordered or they do not conform to an icosahedral symmetry. As a result, the AlphaFold predictions for these regions have low confidence scores. An exception to this observation among the ssDNA viruses is φX174, with its determined CP structure ordered from amino acid 2 and its N-terminus located near the 3-fold axis [48]. However, for the other members of the *Microviridae*, the N-termini are less ordered with, the first observable residue being aa4 to 11 [53,54,55,56]. The major CP N-termini of the *Parvoviridae* often consist of many glycine residues (Figure 13), which is suggested to confer flexibility to its overlapping minor CPs containing a phospholipase domain for externalization from the interior of the capsid [22,102,103]. However, the Aleutian mink disease virus, assigned to the *Amdoparvovirus* genus, lacks a phospholipase domain and has one of the most extensive glycine repeats of the family [104]. Similarly, the capsids of the *Bidnaviridae* have many glycines at their CP N-termini. The N-termini of the remaining ssDNA virus families tend to be very positively charged, with isoelectric points of >10 (Figure 13). Overall, the percentage of basic amino acids in the N-termini ranges from ~20–55% (lowest: *Smacoviridae*; highest: *Pecoviridae*). This region is often also referred to as an arginine-rich motif (ARM), which is capable of DNA binding and thus, plays a role in genome encapsidation [95,96,97,105]. Some of the viruses (e.g., TTMV-Ly1 of the *Anelloviridae*) also contain an increased number of aromatic residues that could assist in DNA interactions via π-stacking [106]. Additionally, the ARM of PCV2 has been described to function as a nuclear localization signal and disruptor of the endosomal membrane following viral entry into the host cell as a substitute to the phospholipase domain of the parvoviruses [107,108]. These functions may also be used by the other ssDNA virus families with similar ARMs.

## 7. Conclusions

The virosphere of ssDNA viruses infects hosts of all kingdoms of life that exist in a wide range of divergent niche environments. As a result, their capsids have evolved to assemble, package genomes, and remain intact under a broad range of conditions until the right stimulus triggers the release of the genome, following the uptake into a suitable host cell, mediated by specific capsid–receptor interactions. Thus, their CPs and, therefore, their assembled capsids have evolved different appearances and sizes to fit their environmental needs. Despite the variable appearances, the viruses’ capsids are assembled from similar core building blocks containing the jelly-roll fold. In different viruses these CPs are arranged with different tessellations to form icosahedral spheres. To date, only eight of the twenty-eight ssDNA virus families with icosahedral capsids have experimentally determined structures. However, the recent advancements in protein folding algorithms have allowed for the prediction of the capsid structures for these remaining families. However, the exact placement of the CPs within the multimeric capsids is difficult to predict, highlighting the need to continue the experimental determination of the capsid structures using cryo-EM. Further structural characterization of the ssDNA capsidome may aid in the design of completely artificial viral capsids or nanoparticles. This can form a blueprint or template for the use of artificial intelligence to optimize viruses as vaccines, gene therapy vectors, and/or cancer immunotherapy agents [109,110,111,112].

## Figures and Tables

**Table 2:** Length of the β-strand connecting loops of the T = 1 ssDNA virus families.

	Loop	*Nano-*	*Circo-*	*Gemini-*	*Anello-*	*Parvo-*	*Micro-*
number of amino acids ^location^		FBNSV	PCV2	MSV	ANV-Ly1	CPV	φX174
	preB	30 *	43 *	40 *	58 *	57 *	9 *
	BC	4 ^5f^	9 ^5f^	12 ^5f^	26 ^5f^	37 ^3/5f^	5 ^5f^
	CD	12 ^3f^	22 ^2f^	17 ^3f^	24 ^2f^	21 *	2 *
	DE	4 ^5f^	9 ^5f^	9 ^5f^	5 ^5f^	26 ^5f^	10 ^5f^
	EF	**26 ^2f^**	24 ^2f^	**36 ^2f^**	26 ^2/5f^	**73** ^2/3f^	**163** ^2/3f^
	FG	8 ^5f^	4 *	22 ^5f^	10 ^5f^	5 *	20 ^5f^
	GH	9 ^2f^	**36** ^3f^	24 ^2f^	20 ^3f^	**229** ^2/3f^	3 *
	HI	3 ^5f^	5 ^5f^	6 ^5f^	**300** ^5f^	21 ^5f^	**110** ^5f^
	postI	2 ^3f^	8 ^2f^	4 *	128 ^2/3f^	45 ^2f^	12 *
	total aa	172	230	244	672	584	426
	% loop	38%	47%	52%	61%	71%	73%

Asterisk indicates that residues are not located on the capsid surface. The 2f, 3f, or 5f superscript indicates the location of the loops on the capsid surface. The gray background indicates the regions preceding the β-strand B or following the β-strand I. Bolded numbers indicate the longest loops.

**Table 3:** Length of the β-strand connecting loops for the T > 1 ssDNA viruses.

		*Bacilladna-*	*Finnlake-*		*Ficleduo-*	
	Loop	CtenDNAV-II	FLiP		φCjT23	
			first	second	first	second
number of amino acids	preB	88	0	17 ^#^	0	7 ^#^
	BC	9	3	6	6	5
	CD	**48**	7	6	4	4
	DE	8	**32**	13	10	5
	EF	**54**	2	4	5	2
	FG	7	**30**	**25**	**23**	**30**
	GH	**53**	18	7	12	4
	HI	12	12	12	7	5
	postI	42	17 ^#^	31	7 ^#^	1
	total aa	390	311		239	
	% loop	49%	62%		54%	

^#^ Indicates the connecting loop between the first and second jelly-roll motif. The gray background indicates the regions preceding the β-strand B or following the β-strand I. Bolded numbers indicate the longest loops.

**Table 4:** Loop lengths of ssDNA viruses with no currently determined capsid structures.

number of aa	virus family		*Nenya-*	*Narya-*	*Kirko-*	*Draupnir-*	*Metaxy-*	*Amesu-*	*Mahapuna-*	*Vilya-*	*Genomo-*	*Ourobo-*
	virus		VVE CG171	EACDV2	DoKiV H46	MCV B19	CFDV	TFDaV	CapV36	GACDV1	HaGvV1	DelV2
	Accession #		YP_010784521	YP_010800608	UJP31654	YP_009121933	AVX29445	AKR53201	QDJ95278	YP_010800614	YP_009181995	QSX73071
	preB		20	23	18	31	32	37	73	59	54	59
	loop	BC	11	5	15	10	7	4	10	14	4	4
		CD	13	12	8	18	13	16	27	15	20	22
		DE	6	11	18	17	10	10	12	8	3	8
		EF	**18**	**18**	**30**	**23**	**39**	**32**	**28**	**37**	**62**	**56**
		FG	6	14	6	5	7	16	3	7	7	16
		GH	9	6	9	**26**	14	12	15	**51**	24	15
		HI	9	7	11	8	9	11	12	11	10	13
	postI		2	2	1	10	1	1	4	3	1	4
	total aa		171	180	188	206	217	237	268	268	284	284
	%loop		42%	41%	52%	52%	46%	43%	40%	53%	46%	47%
number of aa	virus family		*Anicre-*	*Adama-*	*Endolina-*	*Geplana-*	*Peco-*	*Smaco-*	*Kanorau-*	*Redondo-*	*Gandr-*	*Bidna-*
	virus		DflaCV10	DcDSV	ChiDV2	RPfacDV10	HPeCV	ChiSCV2	BfV15	HlaVV	M-ctdb796	BmBDV2
	Accession #		AHH31483	AIY31262	YP_009551343	UBJ26226	YP_009551325	YP_009508861	QCX35050	QCD25321	AXH76206	BAA85361
	preB		80	31	43	110	51	22	122	65	90	74
	loop	BC	4	17	6	5	35	6	8	**76**	5	2
		CD	20	16	31	**48**	**68**	18	**52**	20	**65**	27
		DE	15	6	13	5	11	28	5	**69**	9	20
		EF	23	**63**	21	**48**	**58**	**66**	**57**	**63**	**52**	**146**
		FG	5	37	6	2	6	4	2	4	6	5
		GH	**37**	3	**43**	17	**61**	**173**	18	**84**	**96**	**87**
		HI	6	24	8	17	23	8	31	**54**	14	12
	postI		29	1	54	38	14	29	56	30	57	37
	total aa		290	297	304	386	388	420	443	530	463	386
	%loop		38%	56%	42%	37%	68%	72%	39%	70%	53%	77%

The gray background indicates the regions preceding the β-strand B or following the β-strand I. Bolded numbers indicate the longest loops.

## Supplementary Materials

The following supporting information can be downloaded at: https://www.mdpi.com/article/10.3390/v17030333/s1, Table S1: Summary of deposited *Parvoviridae* capsid structures.
