# Supplementary material for: Structural Capsidomics of Single-Stranded DNA Viruses
Source: Viruses. 2025 Feb 27;17(3):333. doi: 10.3390/v17030333 (PMC11945456; doi:10.3390/v17030333)
Supplement: Supplementary file 1 [file viruses-17-00333-s001.zip › viruses-3457019-supplementary.pdf]

Table S1: Summary of deposited *Parvoviridae* capsid structures.

	Virus	Structure Determination Method	Resolution in Å	PDB-ID
Parvovirinae	***Amdoparvovirus***			
	AMDV	Cryo-EM	2.4	8EP2
	Aveparvovirus			
	RCPV	Cryo-EM	2.7	9N5L
	TuPV	Cryo-EM	2.4	9N5M
	Bocaparvovirus			
	BPV	X-Ray Crystallography	3.2	4QC8
	CnMV	Cryo-EM	2.7	8TU0
	GBoV1	Cryo-EM	2.8	7LNK
	HBoV1	Cryo-EM	2.9	5URF
	HBoV2	Cryo-EM	2.7	7L0V
	HBoV3	Cryo-EM	2.8	5US7
	HBoV4	Cryo-EM	3.0	5US9
	PBoV1	Cryo-EM	2.3	8TU1
	RBoV	Cryo-EM	2.5	8TU2
	Dependoparvovirus			
	AAV1	X-Ray Crystallography	2.5	3NG9
	AAV2	Cryo-EM	2.8	8FYW
	AAV3	X-Ray Crystallography	2.6	3KIC
	AAV4	Cryo-EM	2.2	7THR
	AAV5	Cryo-EM	2.1	7KP3
	AAV6	X-Ray Crystallography	3.0	3OAH
	AAV7	Cryo-EM	2.7	7JOT
	AAV8	Cryo-EM	2.4	9DC2
	AAV9	X-Ray Crystallography	2.8	3UX1
	AAV11	Cryo-EM	2.9	7L6F
	AAV12	Cryo-EM	2.5	7L6B
	AAV13	Cryo-EM	2.8	7L6I
	AAVhu.37	Cryo-EM	2.6	6U95
	AAVhu.69	Cryo-EM	2.5	6U3Q
	AAV-TT	Cryo-EM	3.4	7NA6
	AAVrh.8	X-Ray Crystallography	3.5	4RSO
	AAVrh.10	Cryo-EM	2.8	6O9R
	AAVrh.32.33	X-Ray Crystallography	3.5	4IOV
	AAVrh.39	Cryo-EM	3.4	6V1T
	AvianAAV	Cryo-EM	2.5	8TEX
	BtAAV-10HB	Cryo-EM	3.0	6WFT
	AAVgo.1	Cryo-EM	2.9	7TI4
	AAVpo.1	Cryo-EM	1.7	9N5X
	GPV	Cryo-EM	2.3	9ME0
	SAAV	Cryo-EM	3.3	7U94
	Erythroparvovirus			
	B19	X-Ray Crystallography	3.5	1S58
	Protoparvovirus			
	BuV1	Cryo-EM	2.8	6BWX
	BuV2	Cryo-EM	3.8	6BX0
	BuV3	Cryo-EM	3.3	6BX1
	CPV	X-Ray Crystallography	3.0	2CAS
	CPV	X-Ray Crystallography	2.9	4DPV
	CPV2a	X-Ray Crystallography	3.3	4QYK
	CuV	Cryo-EM	2.9	6X2I
	FPV	X-Ray Crystallography	3.3	1FPV
	H1-PV	X-Ray Crystallography	2.7	4G0R
	LuIII	Cryo-EM	3.2	6B9Q
	MVMi	X-Ray Crystallography	3.5	1Z1C
	MVMp	X-Ray Crystallography	3.3	1Z14
	PPV	X-Ray Crystallography	3.5	1K3V
	TuV	Cryo-EM	2.9	6X2K
	Tetraparvovirus			
	PARV4	Cryo-EM	3.1	8EP9
Densovirinae	***Blattambidensovirus***			
	ZmBWV	Cryo-EM	2.7	8T9C
	Iteradensovirus			
	BmDV	X-Ray Crystallography	3.1	3P0S
	Protoambidensovirus			
	GmDV	X-Ray Crystallography	3.6	1DNV
	Scindoambidensovirus			
	AdDV	X-Ray Crystallography	3.5	4MGU
Hamaparvovirinae	***Brevihamadensovirus***			
	AdSDV	Cryo-EM	2.3	8ER8
	Penstyldensovirus			
	PstDV1	X-Ray Crystallography	2.5	3N7X
	**unassigned**			
	PmMDV	Cryo-EM	3.0	6WH3
